# Development and External Validation of the Korean Prostate Cancer Risk Calculator for High-Grade Prostate Cancer: Comparison with Two Western Risk Calculators in an Asian Cohort

**DOI:** 10.1371/journal.pone.0168917

**Published:** 2017-01-03

**Authors:** Jae Young Park, Sungroh Yoon, Man Sik Park, Hoon Choi, Jae Hyun Bae, Du Geon Moon, Sung Kyu Hong, Sang Eun Lee, Chanwang Park, Seok-Soo Byun

**Affiliations:** 1 Department of Urology, Korea University College of Medicine, Seoul, Republic of Korea; 2 Department of Electrical and Computer Engineering, Seoul National University, Seoul, Republic of Korea; 3 Department of Statistics, College of Natural Sciences, Sungshin Women's University, Seoul, Republic of Korea; 4 Department of Urology, Korea University Ansan Hospital, Ansan, Republic of Korea; 5 Department of Urology, Seoul National University College of Medicine, Seoul, Republic of Korea; 6 Anesthesia Consultants of Indianapolis, Indiana, United States of America; Eberhard Karls University, GERMANY

## Abstract

**Purpose:**

We developed the Korean Prostate Cancer Risk Calculator for High-Grade Prostate Cancer (KPCRC-HG) that predicts the probability of prostate cancer (PC) of Gleason score 7 or higher at the initial prostate biopsy in a Korean cohort (http://acl.snu.ac.kr/PCRC/RISC/). In addition, KPCRC-HG was validated and compared with internet-based Western risk calculators in a validation cohort.

**Materials and Methods:**

Using a logistic regression model, KPCRC-HG was developed based on the data from 602 previously unscreened Korean men who underwent initial prostate biopsies. Using 2,313 cases in a validation cohort, KPCRC-HG was compared with the European Randomized Study of Screening for PC Risk Calculator for high-grade cancer (ERSPCRC-HG) and the Prostate Cancer Prevention Trial Risk Calculator 2.0 for high-grade cancer (PCPTRC-HG). The predictive accuracy was assessed using the area under the receiver operating characteristic curve (AUC) and calibration plots.

**Results:**

PC was detected in 172 (28.6%) men, 120 (19.9%) of whom had PC of Gleason score 7 or higher. Independent predictors included prostate-specific antigen levels, digital rectal examination findings, transrectal ultrasound findings, and prostate volume. The AUC of the KPCRC-HG (0.84) was higher than that of the PCPTRC-HG (0.79, p<0.001) but not different from that of the ERSPCRC-HG (0.83) on external validation. Calibration plots also revealed better performance of KPCRC-HG and ERSPCRC-HG than that of PCPTRC-HG on external validation. At a cut-off of 5% for KPCRC-HG, 253 of the 2,313 men (11%) would not have been biopsied, and 14 of the 614 PC cases with Gleason score 7 or higher (2%) would not have been diagnosed.

**Conclusions:**

KPCRC-HG is the first web-based high-grade prostate cancer prediction model in Korea. It had higher predictive accuracy than PCPTRC-HG in a Korean population and showed similar performance with ERSPCRC-HG in a Korean population. This prediction model could help avoid unnecessary biopsy and reduce overdiagnosis and overtreatment in clinical settings.

## Introduction

The characteristics of prostate cancer (PC) are known to be heterogeneous [1]. Therefore, PC is categorized into groups, such as clinically insignificant or clinically significant, and efforts have been made to differentiate between these groups, because one of the recent issues in PC is overdiagnosis and overtreatment [2]. The European Randomized Study of Screening for Prostate Cancer Risk Calculator (ERSPCRC) has been developed based on 6,288 Dutch participants in the screening arm of the European Randomized Study of Screening for Prostate Cancer study [3]. The official website of ERSPCRC provides the possibilities of both PC of any grade and high-grade (advanced-stage) PC [4]. The Prostate Cancer Prevention Trial Risk Calculator (PCPTRC) was developed based on 5,519 men in the prostate cancer prevention trial, and recently it was revised into version 2.0 [5, 6]. However, it has not been validated in Asian cohorts.

Our team showed in previous studies that the Korean Prostate Cancer Risk Calculator was better than a Western calculator in predicting the probability of PC [7, 8]. Here, we developed the Korean Prostate Cancer Risk Calculator for high-grade prostate cancer (KPCRC-HG) in order to reduce overdiagnosis and overtreatment of PC in our daily practice. This calculator predicts the probability of Gleason score 7 or higher PC at the initial prostate biopsy. We report its development, validation, and comparisons with ERSPCRC for high-grade prostate cancer (ERSPCRC-HG) and Prostate Cancer Prevention Trial Risk Calculator 2.0 for high-grade prostate cancer (PCPTRC-HG).

## Materials and Methods

### Ethics statement

The Institutional Review Board of Korea University Ansan Hospital (Ansan, Republic of Korea) approved this study (approval number: AS 14156–002). The need for informed consent from patients was waived by the Institutional Review Board because this was a retrospective study.

### Model development population and validation population

Data for the model development population, such as patient age, digital rectal examination (DRE) findings, total prostate-specific antigen (PSA) level, transrectal ultrasound (TRUS) findings, prostate volume (PV), and prostate transitional zone volume (TV), were collected from 602 consecutive patients who underwent TRUS biopsy at Korea University medical center between January 2004 and December 2008. These data were identical to those used in the previous studies, and the inclusion/exclusion criteria and the details about TRUS/TRUS biopsy procedure have been described previously [7, 8]. Briefly, biopsy was performed systemically and its indications were increased level of PSA, a palpable nodule upon DRE, or a hypoechoic lesion upon TRUS [7].

DRE was classified as abnormal if there was any prostatic nodule or induration. TRUS findings were classified as abnormal if there was any presence of a hypoechoic lesion. Data for the validation population were collected from 2,313 TRUS biopsy cases from Seoul National University Bundang Hospital treated between January 2009 and December 2014. Patients less than 55 years old were excluded because PCPTRC was created to be applicable to men 55 years or older [9]. The other details about inclusion/exclusion criteria, biopsy indication, and procedure of this population were described in the previous study [10].

### Development of Korean Prostate Cancer Risk Calculator for High-Grade Prostate Cancer

The patient age, DRE findings, total PSA level, TRUS findings, PV and TV were evaluated by logistic regression analyses to detect high-grade PC (Gleason score 7 or higher) [11]. In all analyses, the PSA level, PV and TV were natural log-transformed to approximate a normal distribution. Significant variables detected by simple analysis (p-value of <0.05) were included in the multiple logistic regressions with a backward elimination method. The prediction equation predicting the probability of high-grade PC was developed using this final multiple logistic regression analysis.

### Validation and head-to-head comparison with ERSPCRC-HG and PCPTRC-HG

KPCRC-HG was externally validated using the validation cohort regarding predictive accuracy and performance characteristics with receiver operating characteristic curves and calibration plots, respectively. The area under the receiver operating characteristic curve (AUC) was calculated for the KPCRC-HG, ERSPCRC-HG, and PCPTRC-HG using the validation cohort. The calculated probabilities of ERSPCRC-HG were provided by Dr. Roobol. The logit1 of PCPTRC-HG was calculated as −3.002 + 0.256 × log_2_(PSA) + 0.016 × age + 0.122 × race − 0.455 × prior biopsy − 0.039 × DRE + 0.272 × family history, and logit2 was −7.053 + 0.705 × log_2_(PSA) + 0.048 × age + 1.042 × race − 0.214 × prior biopsy + 0.401 × DRE + 0.225 × family history [6]. If the DRE or prior biopsy was positive or there was a family history of PC, the value of these variables was 1; otherwise, the value was 0. All the people in the validation cohort were Asian, so race in these logits was equal to 0. The probability function of PCPTRC-HG was calculated as exp (logit2) / [1+ exp (logit1) + exp (logit2)]. The statistical significance of differences in ROC curve areas was determined by the methods of Hanley and McNeil [12]. Calibration was assessed by grouping patients into 40 groups (each comprising 57 or 58 patients in the external validation cohort) with respect to their predicted probabilities and then comparing the mean of each group with the observed proportion of men with cancer [13]. The sum of squares of the residuals (SSR) was calculated to evaluate the deviation from perfect prediction (the 45° line).

Lastly, the number of biopsies saved, positive predictive value (PPV), negative predictive value (NPV), and high-grade PC lost according to the threshold probability of these calculators were counted and compared with the expected number if a PSA-based decision (cut-off ≥ 4.0 ng/mL) was applied to the cohort. The number of biopsies saved means the number of biopsy cases with a negative result or a positive result with Gleason score 6 or less where the predicted probability is below the threshold probability, and it implies that this number of patients could have avoided unnecessary TRUS biopsy if the calculator were applied.

All statistical outcomes were presented as follows: continuous variables were expressed as either the mean ± standard deviation (SD), median [inter-quartile range], or numbers (percentage) of cases, and the odds ratio and the 95% confidence interval. Categorical variables were reported as the number of occurrences and frequency. Student’s t-test and the Pearson χ2 test were used for statistical comparisons of continuous and categorical variables, respectively. All statistical analyses were performed using SPSS version 20.0 or R for Windows, version 3.0.1 (http://www.r-progect.org/). We regarded a p-value <0.05 as statistically significant.

## Results

The characteristics of the development and validation cohorts are listed in Table 1. In the model development cohort, PC was diagnosed in 172 men (28.6%), with 120 (19.9%) of them being Gleason score 7 or higher. The assigned Gleason score was 2–4 in 5 cases (2.9%), 6 in 47 cases (27%), 7 in 35 cases (20%), and 8–10 in 85 cases (50%) among the cases of PC. All of the variables listed above were statistically significant predictors of high-grade PC upon needle biopsy (all p < 0.05) in the simple logistic regression analysis (Table 2). In the multiple logistic regression analysis with an enter method, the significant predictors were DRE findings, TRUS findings, the logarithmic transformations of PSA level, and the logarithmic transformations of PV (Table 2). The logit of this prediction model is calculated as 2.308 + 0.515 × DRE + 0.904 × TRUS + 1.446 × ln(PSA)-1.902 × ln(PV). The probability function was calculated as exp (logit) / (1+ exp [logit]) [14]. For continuous variables, such as PSA and PV, the value itself was put into the equation. For the categorical variable of DRE findings or TRUS findings, 0 was used in the equation when normal, and 1 was used when abnormal. Using this equation, we developed the KPCRC-HG, which predicts the probability of high-grade PC (Gleason score 7 or higher) in Korean men. It is available at the following website: http://science.aci-llc.net/prostate. The predictive accuracy of this calculator was 0.91 (95% CI, 0.88–0.95) calculated by AUC in the internal cohort.

**Table 1:** The clinical characteristics of the development cohort and validation cohort.

Variables	Development cohort	Validation cohort
**Patients (n)**	602	2313
**Age (years) (mean ± SD)**	65.7 ± 9.1	67.7 ± 6.84
**PSA (ng/mL) (median [inter-quartile range])**	6.77 [4.41–12.19]	7.33 [4.66–13.35]
**Prostate size (ml) (median [inter-quartile range])**	38.7 [28.4–52.6]	39.0 [30.0–53.0]
**Transitional zone size (ml) (median [inter-quartile range])**	17.1 [10.6–27.0]	17.0 [11.0–27.0]
**Nodule by DRE (n) (%)**	149 (24.8)	577 (24.9)
**Abnormal TRUS finding (n) (%)**	241 (40.0)	448 (19.4)
**Cancer detection (n) (%)**	172 (28.6)	989 (42.8)
**Gleason sum ≥ 7 (n) (%)**	120 (19.9)	614 (26.5)

PSA, prostate-specific antigen; DRE, digital rectal examination; TRUS, transrectal ultrasound

**Table 2:** Simple and multiple logistic regression analyses in the development cohort.

	Simple logistic regression analysis			Multiple logistic regression analysis		
	OR	95% CI	P value	OR	95% CI	P value
**Age**	1.06	1.03–1.09	<0.001			
**ln PSA**	4.46	3.40–5.85	<0.001	4.25	3.63–4.98	<0.001
**ln PV**	0.52	0.33–0.83	0.006	0.15	0.11–0.20	<0.001
**ln TV**	0.61	0.45–0.84	0.002			
**Nodule by DRE**	7.51	4.85–11.6	<0.001	1.67	1.30–2.16	<0.001
**Abnormal TRUS finding**	5.34	3.43–8.29	<0.001	2.47	1.88–3.26	<0.001
**Intercept**				10.052		

PSA, prostate-specific antigen; PV, prostate volume; TV, prostate transitional zone volume; DRE, digital rectal examination; TRUS, transrectal ultrasound

**Fig 1:**
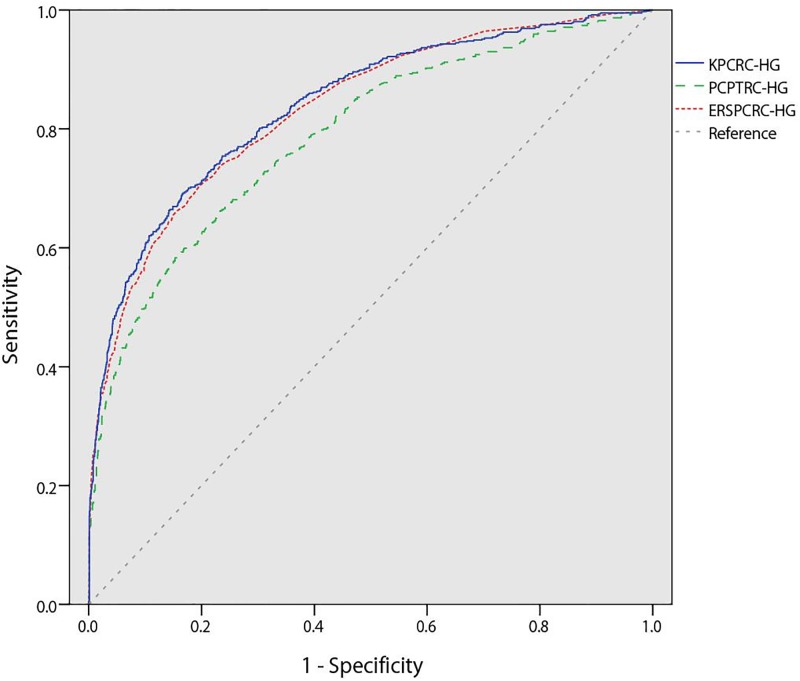
Receiver operating characteristic curves of the Korean Prostate Cancer Risk Calculator for High-Grade Prostate Cancer (KPCRC-HG), the European Randomized Study of Screening for PC Risk Calculator for high-grade cancer (ERSPCRC-HG), and the Prostate Cancer Prevention Trial Risk Calculator 2.0 for high-grade cancer (PCPTRC-HG).

**Fig 2:**
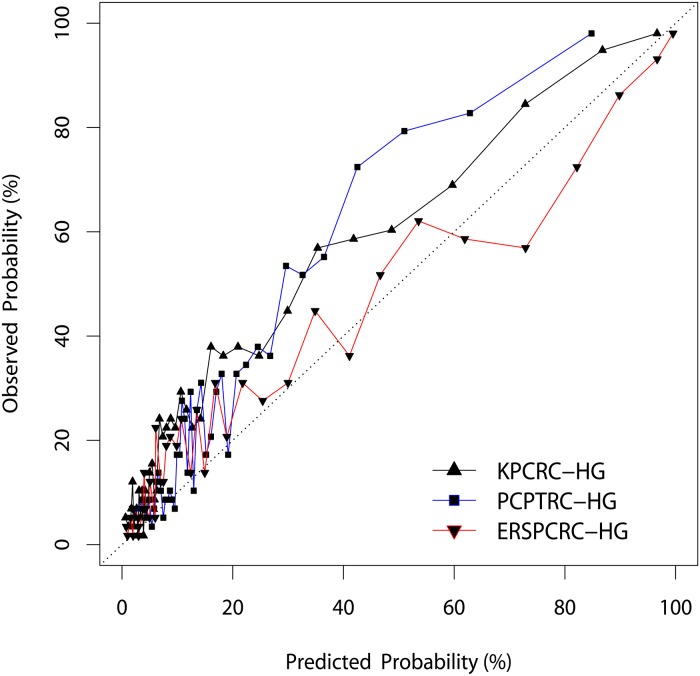
Calibration plots depicting the agreement between predicted and observed probabilities of positive biopsy of the Korean Prostate Cancer Risk Calculator for High-Grade Prostate Cancer (KPCRC-HG), the European Randomized Study of Screening for PC Risk Calculator for high-grade cancer (ERSPCRC-HG), and the Prostate Cancer Prevention Trial Risk Calculator 2.0 for high-grade cancer (PCPTRC-HG).

In the external validation cohort, PC was diagnosed in 989 men (42.8%), with 614 (26.5%) of these being Gleason score 7 or higher. KPCRC-HG, ERSPCRC-HG, and PCPTRC-HG were externally validated with this cohort. The AUC of the KPCRC-HG (0.84; 95% CI, 0.82–0.86) was significantly higher than that of PCPTRC-HG (0.79; 95% CI, 0.77–0.81, p<0.001) but not different from that of ERSPCRC-HG (0.83; 95% CI, 0.81–0.85) (Fig 1). In addition, KPCRC-HG showed overall better calibration than did PCPTRC-HG but it did not show better calibration than did ERSPCRC-HG (SSR of 0.50 for the KPCRC-HG, 0.60 for PCPTRC-HG and 0.21 for ERSPCRC-HG) (Fig 2). Taken together, both KPCRC-HG and ERSPCRC-HG showed similar predictive accuracy, and their performance as prediction models was better than that of PCPTRC-HG.

Table 3 shows the number of biopsies saved, PPV, NPV, and high-grade PC lost at the threshold probabilities of 3, 5, 10, and 15% using these calculators, along with the missed cancers if a PSA-based decision was undertaken in the validation cohort. At the threshold probability of 7% of KPCRC-HG (numbers of biopsies were 1,782), the number of biopsies saved, PPV, NPV, and high-grade PC lost were better compared to those achieved when a PSA-based decision was undertaken. With a PSA cut-off level set at ≥4.0 ng/mL, 1,892 patients would have undergone biopsy. However, the number of missing high-grade cancer was 31, which was higher than the 23 achieved when using a threshold probability of 7% with the KPCRC-HG. Considering that this calculator was invented to detect high-grade PC, a threshold probability value of 5% in KPCRC-HG seems to be proper in this validation cohort to minimize the number of missing cases.

**Table 3:** Comparison of diagnostic accuracy at various threshold probabilities of risk calculators and of a PSA cut-off level of 4.0 ng/mL.

Threshold probability (%)	Biopsies, no. (A)	Biopsies saved, no. (B = 2313-A-D) (%, B/2,313)	High-grade PC detected, no. (C)	PPV (%, C/A)	NPV (%, B/(2313-A))	High-grade PC lost, no. (D = 614-C) (%, (614-C)/614)
**5**						
**KPCRC-HG**	2046	253 (10.9)	600	29.3	94.8	14 (2.3)
**ERSPCRC-HG**	1,634	643 (27.8)	578	35.4	94.7	36 (5.9)
**PCPTRC-HG**	2102	197 (8.5)	600	28.5	93.4	14 (2.3)
**7**						
**KPCRC-HG**	1853	437 (18.9)	591	31.9	95.0	23 (3.7)
**ERSPCRC-HG**	1390	860 (37.2)	551	39.6	93.2	63 (10.3)
**PCPTRC-HG**	1824	450 (19.5)	575	31.5	92.0	39 (6.4)
**10**						
**KPCRC-HG**	1552	719 (31.1)	572	36.9	94.5	42 (6.8)
**ERSPCRC-HG**	1,146	1,066 (46.1)	513	44.8	91.3	101 (16.4)
**PCPTRC-HG**	1,398	836 (36.1)	535	38.3	91.4	79 (12.9)
**15**						
**KPCRC-HG**	1191	1,036 (44.8)	528	44.3	92.3	86 (14.0)
**ERSPCRC-HG**	907	1,254 (54.2)	462	50.9	89.2	152 (24.8)
**PCPTRC-HG**	901	1,225 (53.0)	427	47.4	86.8	187(30.5)
**PSA cut-off ≥ 4.0 ng/mL**	1,892	390 (16.9)	583	30.8	92.6	31 (5.0)
**Total**	2,313		614	26.5		

no., numbers; PC, prostate cancer; PPV, positive predictive value; NPV, negative predictive value; KPCRC-HG, Korean Prostate Cancer Risk Calculator for high-grade prostate cancer; ERSPCRC-HG, European Randomized Study of Screening for PC Risk Calculator for high-grade prostate cancer; PCPTRC-HG, Prostate Cancer Prevention Trial Risk Calculator 2.0 for high-grade prostate cancer; PSA, prostate-specific antigen

## Discussion

Serum PSA level is associated with PC diagnosis, prognosis, and treatment response [15]. However, PSA levels depend on other clinical factors, such as age, inflammation, and prostate size [16, 17]. Accordingly, many efforts were made to develop prediction models for PC based on clinical, laboratory, and/or ultrasound parameters in order to improve the rates of prostate cancer detection [18, 19]. Many prediction models based on Western data to diagnose PC at initial biopsy have been developed and compared each other [4, 5]. Our team also developed a prostate cancer risk calculator based on a Korean cohort, validated it externally, and compare its performance with Western risk calculators [7, 8]. However, unlike the ERSPCRC-HG and PCPTRC-HG, our previous calculator does not predict high-grade PC. That is the reason why we invented and validated this new calculator.

The significant factors in KPCRC-HG are DRE findings, TRUS findings, PSA level, and PV, which are just the same with those in ERSPCRC-HG and those in PCPTRC-HG are age, DRE findings, and PSA. The other variables in the PCPTRC-HG, such as race, family history, and prior biopsy, played no role in calculating probabilities of our prediction model, because all participants were Asian with no prior biopsy, and our database did not include information on family history. Most variables mentioned above are known to be related to high-risk PC, though age has not been proven to be associated with high-risk PC so far [5, 20, 21]. For this reason, KPCRC-HG and ERSPCRC-HG might perform better than PCPTRC-HG. Our previous study demonstrated that KPCRC predicting the possibility of PC with low- to high-grade performed better than ERSPCRC in a Korean population [8]. By contrast, in the present study, KPCRC-HG and ERSPCRC-HG were not so different in predicting the possibility of a high-grade PC diagnosis. This result may come from the fact that these 2 calculators have the same predicting factors. We assume that the characteristics of PC are different among races, whereas the characteristics of high-grade PC may have something in common. However, it has to be confirmed by further investigation. Lastly, we should pay attention that the numbers of “High grade PC lost” in the Table 3 are different between KPCRC-HG and ERSPCRC-HG even though the variables of these 2 calculators are the same. It implies that each population should have its own predictor for the certain disease risk.

After the ERSPC and Prostate Lung Colorectal Ovarian Cancer screening trials reported overdetection and overtreatment of PC, many researchers have questioned the need for community screening with PSA and DRE [22, 23]. A recent study from a database consisting of 2,411 consecutive patients undergoing radical prostatectomy reported that the risk of clinically insignificant disease was found to be 31.1% [24]. Since the morbidities from TRUS biopsy and PC treatment are significant, avoiding biopsy in men at lower risk would be ideal. Careful patient selection for screening and reducing overtreatment are important to preserve the benefits and reduce the harms of PSA testing. Notably, this must be considered carefully when using these data to make policy, because all of these estimates are extremely population-based and context-specific.

The old-fashioned aim several decades ago was to detect as many patients with PC as possible. Nowadays, most guidelines recommend detecting and treating only clinically significant PC [21, 25]. Moreover, active surveillance has recently emerged as a primary management strategy in men with favorable-risk PC [26]. Therefore, if one can predict clinically insignificant PC before prostate biopsy, adverse effects, and the medical cost of prostate biopsy could be avoided. Several studies have been performed to distinguish clinically significant PC. The Prostate Health Index combining total, free, and [-2]proPSA into a single score had greater predictive accuracy for clinically significant prostate cancer, leading to its recent FDA approval as an aid to PC detection for men with a PSA of 4 to 10 ng/ml [27]. The Prostate Health Index was also shown to be useful to predict reclassification during active surveillance of PC patients [28]. Multiparametric magnetic resonance imaging has been regarded as another tool for detecting clinically significant PC. A recent systematic review on the diagnostic accuracy of multiparametric magnetic resonance imaging reported that the detection rate of clinically significant PC ranged from 44% to 87%, and the negative predictive value for exclusion of significant disease ranged from 63% to 98% [29]. In order to rule out significant disease, the high negative predictive value would be important to the clinician. In the present study, when the threshold probability value of KPCRC-HG was 3%, the negative predictive value was 94.5%, implying that KPCRC-HG can play a role in excluding significant disease before biopsy.

The American Urological Association guidelines, revised in 2013, suggest that risk calculators predicting the risk of PC have not been proven its efficacy and that their value in predicting cancer on biopsy is not necessarily applied to a population that differs from that in which the calculator was derived [30]. In the National Comprehensive Cancer Network guidelines, the panel does not recommend the use of risk calculators alone to determine whether to biopsy, but also indicates that these calculators have as much value in determining who might not need biopsy as in identifying those at high risk [21]. In the European Association of Urology guidelines revised in 2016, the panel recommended the use of risk calculators for asymptomatic men with a PSA between 2–10 ng/mL prior to performing a prostate biopsy, because these are useful in helping to determine what the potential risk of PC may be, and thereby reducing the number of unnecessary biopsies [25]. Since PSA is not a perfect measure for PC detection at this moment, additional testing would be inevitable in this clinical setting.

A limitation of our study is the use of biopsy Gleason score to define clinical significance. Biopsy Gleason score may be changed if the patient undergoes radical prostatectomy. KPCRC-HG had similar performance with ERSPCRC-HG in the external validation cohort. One might ask whether another risk calculator is required when it is not better than the original one. However, no one can know the clinical value of the prediction model before external validation, which we did in this study. Through further investigation with more data included, an upgraded calculator can be invented in the future. There are several advantages in the present study. The prediction model was developed and validated using a large-scale cohort. Since this model was based on common clinical information, it has a lower level of complexity. This study reports the development of KPCRC-HG as well as its validation and head-to-head comparison with Western internet web-based risk calculators.

## Conclusions

We developed KPCRC-HG for predicting the probability of high-grade PC using data from a Korean male cohort, and compared its performance with ERSPCRC-HG and PCPTRC-HG. KPCRC-HG had higher predictive accuracy than did PCPTRC-HG, while showing a similar performance to ERSPCRC-HG in a Korean population. If KPCRC-HG is used in a clinical setting, it will provide meaningful information for physicians and patients during personalized shared decision-making for TRUS biopsy. Furthermore, it could help in avoiding unnecessary biopsy and reducing overdiagnosis and overtreatment. When validated in other Asian countries, KPCRC-HG may also have the potential to be used for other Asian populations.

## Supporting Information

S1 File Minimal data set for this study. (XLS)
