# Correction: Development and External Validation of the Korean Prostate Cancer Risk Calculator for High-Grade Prostate Cancer: Comparison with Two Western Risk Calculators in an Asian Cohort

**DOI:** 10.1371/journal.pone.0175606

**Published:** 2017-04-06

**Authors:** The PLOS ONE Staff

The Funding section is incorrect. The correct Funding information is: This work was supported by the Korea University Grant 2016.

The URL in the first sentence of the “Purpose” section of the Abstract is incorrect. The correct URL is: http://science.aci-llc.net/prostate.

The publisher apologizes for the errors.

## References

[1] Park JY, Yoon S, Park MS, Choi H, Bae JH, Moon DG, et al (2017) Development and External Validation of the Korean Prostate Cancer Risk Calculator for High-Grade Prostate Cancer: Comparison with Two Western Risk Calculators in an Asian Cohort. PLoS ONE 12(1): e0168917 doi:10.1371/journal.pone.0168917 28046017 10.1371/journal.pone.0168917 PMC5207506

